# Innate Immune Components That Regulate the Pathogenesis and Resolution of hRSV and hMPV Infections

**DOI:** 10.3390/v12060637

**Published:** 2020-06-12

**Authors:** Catalina A. Andrade, Gaspar A. Pacheco, Nicolas M. S. Gálvez, Jorge A. Soto, Susan M. Bueno, Alexis M. Kalergis

**Affiliations:** 1 Millennium Institute of Immunology and Immunotherapy, Departamento de Genética Molecular y Microbiología, Facultad de Ciencias Biológicas, Pontificia Universidad Católica de Chile, Santiago 8320000, Chile; cnandrade@uc.cl (C.A.A.); grpacheco@uc.cl (G.A.P.); nrgalvez@uc.cl (N.M.S.G.); jasoto6@uc.cl (J.A.S.); sbueno@bio.puc.cl (S.M.B.); 2 Departamento de Endocrinología, Facultad de Medicina, Pontificia Universidad Católica de Chile, Santiago 8320000, Chile

**Keywords:** hRSV, hMPV, innate immune response, vaccines, immunotherapy

## Abstract

The human respiratory syncytial virus (hRSV) and human Metapneumovirus (hMPV) are two of the leading etiological agents of acute lower respiratory tract infections, which constitute the main cause of mortality in infants. However, there are currently approved vaccines for neither hRSV nor hMPV. Moreover, despite the similarity between the pathology caused by both viruses, the immune response elicited by the host is different in each case. In this review, we discuss how dendritic cells, alveolar macrophages, neutrophils, eosinophils, natural killer cells, innate lymphoid cells, and the complement system regulate both pathogenesis and the resolution of hRSV and hMPV infections. The roles that these cells play during infections by either of these viruses will help us to better understand the illnesses they cause. We also discuss several controversial findings, relative to some of these innate immune components. To better understand the inflammation in the lungs, the role of the respiratory epithelium in the recruitment of innate immune cells is briefly discussed. Finally, we review the main prophylactic strategies and current vaccine candidates against both hRSV and hMPV.

## 1. Introduction

The human respiratory syncytial virus (hRSV), recently renamed human orthopneumovirus, is the leading viral agent that causes acute lower respiratory tract infections (ALRTIs), making this virus the main etiological viral agent by which infants under the age of five die [1,2,3]. It has been reported that hRSV-caused ALRTIs in infants can reach nearly 33 million cases per year, of which 3.2 million require hospitalization, and 59,600 of the hospitalized cases, unfortunately, resulted in the deaths of the infected infants [4]. The economic cost associated with hospitalizations can reach USD 394 million per year, making this virus a serious health care matter [5].

Infection by hRSV affects young children more severely, as well as the immunocompromised and the elderly [6,7]. Infections by this virus usually take place during late fall, winter, and early spring, and can produce symptoms, such as coughing, dyspnea, feeding difficulties, hypoxemia, and wheezing, and this infection can lead to a more severe pathology involving bronchiolitis and pneumonia [8,9]. Additionally, it has been observed that hRSV RNA or proteins can be detected in the central nervous system (CNS), which has been associated with neuro-pathologies, such as esotropia, seizures, and encephalopathies [10,11,12]. Reinfections of this virus occur frequently throughout the lives of the infants, due to the inefficient development of immunological memory produced by the first infection with hRSV. Even though a less severe disease with fewer clinical complications is caused by the reinfection with this virus, cases with lower respiratory tract disease have been reported [13,14].

Even though hRSV was first identified over 60 years ago and has been widely studied ever since its discovery, no safe and effective vaccine has been approved to prevent the disease caused by this virus [15,16].

The human Metapneumovirus (hMPV) is another major viral agent that causes ALRTIs, making it an important pathogen that contributes significantly to the mortality of infants under the age of five [1,2]. A report made in the United States (US) showed that the numbers of outpatient visits are 55/1000, and the cases of children infected by hMPV that needed hospitalization represents 1/1000 children, lower than the 3/1000 cases that hRSV is responsible for, estimating that 20,000 children are hospitalized per year in the US due to hMPV infection [17]. The hospitalization costs associated with this virus are nearly USD 277 million per year, making hMPV an important health care problem as well [18].

hMPV infections cause more complications to young children, the immunocompromised, and elderly patients [19]. hMPV infection is characterized by symptoms, such as cough, rhinitis, fever, wheezing, and hyperventilation, and can lead to a more severe pathology involving bronchiolitis and pneumonia [9]. Additionally, it has been reported that hMPV can produce alterations in the CNS, resulting in seizures and encephalopathies [11,20]. Infection by this virus has been observed to take place during the entire year, but there is generally a peak during the winter or spring seasons. Furthermore, it can cause reinfections throughout the lives of the infants [8,21].

hMPV was discovered more recently than hRSV in 2001, and similar to the latter, no vaccine against hMPV has been approved yet [22,23].

Even though both of these viruses are closely related and share similar symptoms, infection with hRSV causes more cases of bronchiolitis and fewer cases of pneumonia as compared to hMPV [24]. Moreover, immune cell types involved in the resolution of each infection also vary—the immune response elicited in the host by these viruses is responsible for some differences in the symptoms [24,25]. Because of this, along with the fact that the immune responses produced against these viruses are not the same, it is important to characterize the specific innate immune responses for each of these viruses, and their contributions to pathogenesis.

In this article, we will discuss the general characteristics of these viruses along with the innate immune response that these pathogens induce in the host. This work will be focused on dendritic cells (DCs), alveolar macrophages (AMs), neutrophils, eosinophils, natural killer (NK) cells, innate lymphoid cells (ILCs), and the complement system. Additionally, we will discuss how this response can contribute to pathogenesis and/or disease resolution. How the pulmonary epithelium can contribute to both pathogenesis and the recruitment of these immune cells will also be addressed. Current and prospective prophylactic approaches, such as vaccines and monoclonal antibodies that can help to control the infection caused by these viruses, will also be considered. Lastly, we will discuss the similarities and the differences in the immune responses elicited by hRSV and hMPV infections.

## 2. General Features of These Two Respiratory Viruses

hRSV and hMPV belong to the same taxonomical family of viruses, but due to individual characteristics in their genomes, they are classified in different genera within this family. In the following section, the general characteristics of these viruses regarding their genetics and life cycles will be discussed.

### 2.1. Human Respiratory Syncytial Virus (hRSV)

#### 2.1.1. Viral Genes and Structure

hRSV is an enveloped, single-stranded, non-segmented, negative-sensed RNA virus that belongs to the *Pneumoviridae* family, *Orthopneumovirus* genus [3]. Its genome is 15.2 kb in length and contains 10 genes that code for 11 proteins in the following order: 3′-NS1-NS2-N-P-M-SH-F-G-M2-L-5′ [3]. It is noteworthy that the proteins M2-1 and M2-2 are two distinct proteins, a product of the transcription of two different open reading frames (ORFs) of the *m2* gene [3].

The envelope of hRSV contains three proteins on the surface: the glycoprotein (G), the fusion protein (F), and the short hydrophobic protein (SH). The G protein is a heavily glycosylated glycoprotein involved in the attachment of the virus to the target cell via the binding of heparin and/or annexin II on the cell surface [26,27]. As for the F protein, most of the evidence suggests that it binds to the receptor nucleolin [28,29]. This binding mediates the fusion between the viral envelope and the cell membrane, as well as cell–cell fusion, leading to syncytia formation. Similar to other fusion proteins, the F protein exists in two distinct conformational states (pre-fusion and post-fusion) [30,31], which are relevant for the humoral response elicited against this viral antigen, and the exposure of the epitopes that these antibodies recognize [31]. Such transition is presumably triggered by the interaction between F and its receptor nucleolin and is required to bring the viral envelope and the cell membrane closer together to induce the fusion of both [32]. Lastly, the SH protein is a small protein that is expressed on the membranes of infected cells, and is not essential for virus attachment or fusion [33], but rather acts as a viroporin on the surface of infected cells [34,35].

The genome of hRSV is associated with the nucleoprotein (N), the phosphoprotein (P), and the viral RNA-dependent RNA polymerase (L) to form the ribonucleoprotein complexes (RNPs). The main functions of the N protein are to coat the viral RNA in a left-handed helical nucleocapsid to protect it from mechanical, chemical, and physical damage [36,37], and to participate in the replication of the viral genome [38,39]. The P protein is an essential factor for the replication and transcription of the viral genome and is also implicated in the packaging in the nucleocapsid [40,41]. The L protein is responsible for the synthesis of a positive-sensed antigenome that serves as a template for replication, and the transcription of the viral genome into mono- and polycistronic mRNAs [42,43]. The efficient transcription of long polycistronic mRNAs requires the M2-1 protein, since it serves as an anti-termination factor [44] and the M2-2 protein is used as a cofactor necessary for the fine-tuning of gene expression [45].

Matrix proteins M and M2-1 are also present in the virion as structural components [46,47]. The M protein in particular is a bridge between the RNPs and the lipid bilayer envelope. It also serves as an inhibitor of virus transcription in the late stages of infection and facilitates virion assembly and budding by coating the RNPs [48] and modifying the actin cytoskeleton [49].

Lastly, hRSV possesses two non-structural proteins, NS1 and NS2, which are expressed in the early stages of replication. These proteins are considered to be major virulence factors of hRSV since they play an important role in the inhibition of type I IFN expression, thus promoting viral replication and spread to neighboring cells [50,51,52].

#### 2.1.2. Infectious Cycle

hRSV is able to infect bronchial respiratory epithelia. Interestingly, it has been shown that it can also infect neurons in vitro [12,53], as well as DCs inhibiting their capacity to activate T cells by preventing immunological synapse assembly [54,55]. To infect a target cell, hRSV must trigger a two-step entry process involving the electrostatic attachment of the viral particle to the cell membrane through the G protein and the subsequent fusion of both the viral envelope and the cell membrane through the F protein. The G protein is not completely essential for infection to occur, but it facilitates viral entry [33,56]. After viral and host membranes have been fused, the viral contents of hRSV are released into the cell cytoplasm. The uncoating of the genome takes place and the replication and transcription of the viral genome begin. The N, P, L, M2-1, and M2-2 proteins participate in these processes. The M2-2 protein also acts as a regulatory element in the transcription and replication of the viral genome [57]. Finally, the matrix protein (M) is key for understanding the formation of inclusion bodies, virion assembly, and budding, although it has also been observed that it contributes to cell cycle arrest in infected lung epithelial cells [58].

Interestingly, SH protein has been shown to act as a viroporin, assembling into pentameric pores in infected cells [57,58], thus altering the permeability of the membrane to cations [34,35]. Additionally, this protein can inhibit both apoptosis and TNF-α signaling [59], and may be implicated in downregulating the IL-1β response [60], suggesting a role as an important virulence factor. Besides, the SH protein could contribute to the replication and infection process [34].

Lastly, non-structural proteins 1 and 2 (NS1 and NS2) are encoded by the first two genes of hRSV and play crucial roles as virulence factors in early time points of infection [61]. Both of these proteins are able to partly inhibit hRSV-mediated and TNF-α-mediated cellular apoptosis in the early stages of infection [62]. Moreover, they interfere with key viral recognition signaling pathways, downregulating IRF3 and STAT2 expression, thereby limiting the type I interferon (IFN-I) response [50,51].

### 2.2. Human Metapneumovirus (hMPV)

#### 2.2.1. Viral Genes and Structure

hMPV is an enveloped, non-segmented, single-stranded, and negative-sensed RNA virus, which belongs to the genus *Metapneumovirus* of the family *Pneumoviridae* [63,64]. The genome of this virus is 13.3 kb of length and is constituted by eight genes that encode for nine proteins, whose genomic order is the following: 3′-N-P-M-F-M2-SH-G-L-5′. Each of these genes encodes a single protein, except for the *m2* gene, which codes for two different proteins. M2-1 and M2-2 are the result of the overlapping of two ORFs located in the mRNA from the *m2* gene [64]. The genome of hMPV differs from the genome of hRSV in the lack of the NS1 and NS2 proteins.

The hMPV virion consists of an envelope that has three glycoproteins on the surface: the small hydrophobic protein (SH), the attachment protein (G) and the fusion protein (F). The SH protein is a transmembrane protein of type II, which is capable of inhibiting the transcriptional activity of NF-κB, resulting in a decrease in the transcription of genes that use this pathway [65,66]. It has been reported that the SH protein can play an important role in the cycle of the virus through the modulation of the permeability of the membrane [65]. The G protein is a type II transmembrane protein that can bind to the cellular glycosaminoglycans (GAGs), which are found in the membranes of the cells, promoting the attachment of the virus to the cell and playing a role in the infection capability of hMPV [67]. The F protein is a type I fusion protein that is synthesized in the form of an inactive precursor (F0), and, in order to be biologically activated, a cleavage is needed between the subunits F1 and F2, which are connected through disulphide bonds [68]. This protein mediates the attachment and the fusion of the viral envelope of hMPV with the cell membrane to promote the entry of the virus into the cell to be infected.

These glycoproteins found on the surface are associated with the matrix protein (M), which is also connected with the inner membrane from the surface. The M protein interacts with various components from the virus and the cells, promoting the assembly and budding of the virus [69]. Additionally, the M protein can inhibit the transcription of the virus before it is packaged [70].

hMPV RNA is united with the nucleoprotein (N), the phosphoprotein (P), the large polymerase protein (L), the putative transcription factor (M2-1), and the RNA synthesis regulatory factor (M2-2), forming the nucleocapsid. The association between two domains from the N protein and the virus RNA causes the helical nucleocapsid form and protects the genome from any damage [71,72]. The N protein interacts with the P protein, inducing the recruitment of the L protein along with the M2-1. The P protein makes the assembly of the ribonucleoprotein (RNP) complex possible, due to the stabilization of the L protein. Both the P and L proteins conform to the RNA-dependent RNA polymerase (RdRp), and position themselves in the 3′ end of the viral genome to begin the transcription process [72,73].

#### 2.2.2. Infectious Cycle

Ciliated epithelial cells are the primary target of the hMPV infection, and then the virus spreads to all the cells of the respiratory tract where viral replication takes place [74]. The replication process begins in the upper respiratory tract and can spread to the lower respiratory tract, where it can reach the bronchioles and the alveoli. In these structures, the virus has shown higher replication capacities, as compared to the upper respiratory tract [74]. Within the immune cells that can get infected by hMPV, DCs are one of the most relevant, although viral replication is not efficient in these cells [75].

Once the virus arrives at the airway epithelia, the F and G proteins promote the attachment to the airway epithelial cell (AEC), where there is an interaction between the F protein and αvβ1 integrin, along with an interaction between the G protein and GAGs [67,76,77]. When the fusion of the membranes is accomplished, a fusion pore is formed and the hMPV RNP complex can enter into the cytoplasm of the cell to be infected. In the cytoplasm, the N, P, and L proteins detach from the viral RNA and arrange themselves to create the polymerase complex [76]. Additionally, proteins N and P are the only proteins needed to generate inclusion bodies that appear as a result of the replication process [78]. Importantly, the M2-2 protein plays a role in the evasion of the immune response elicited against hMPV infection, inhibiting the expression of genes associated with the MyD88 pathway [79]. This contributes to the spreading of the infection.

hMPV exhibits two phases of replication, characterized by their kinetics of infection: an acute phase of infection with a peak of viral titer 7 days post-infection (d.p.i.) and a second phase of infection with a peak of viral titer at 14 or 28 d.p.i., depending on the virus strain. Of note, the genetic material of hMPV can be found up to 180 d.p.i. within the lungs of BALB/c mice [80,81].

## 3. Differential Regulation of the Innate Immune Response by Each Virus

The innate immune response is the first line of defense against virtually all kinds of pathogens, including those of viral natures. Within this complex response, innate immunity plays a key role in the control of infections given its fast and antigen non-specific responses against pathogens. The most relevant innate immune response components in the contexts of hRSV and hMPV infections are neutrophils, eosinophils, macrophages, dendritic cells, natural killer cells, and the complement system. How each of these cells are activated and the immune pathology, or contribution for disease resolution that results from their recruitment, is discussed below.

### 3.1. Dendritic Cells

Dendritic cells (DCs) are considered part of the first line of defense against viruses and, most importantly, are considered to orchestrate both the innate and the adaptive immune response [82]. DCs can be found in the lung epithelium and are capable of responding quickly during pathogen-caused inflammation [83]. Moreover, DCs are classified into two conventional subtypes (cDC1 and cDC2) and a plasmacytoid one (pDC), of mixed lymphoid and myeloid ontogeny [84,85,86].

During a hRSV infection, all DCs subtypes have been shown to migrate to the lung as early as 2 d.p.i. [87,88], and peak migration occurs at 6 d.p.i. [88]. Importantly, DCs can serve as an important IFN-I sources upon encounter with hRSV [89], although there is one controversial report that found no IFN-I secretion in either DC subset in the lungs of infected mice [90]. This last study showed that only pDCs—among all classically defined DC subsets—contributed minimally to IFN-I secretion. The authors used an *Ifna6*^gfp/+^ mouse strain to define which immune cells contributed to IFN-I secretion and validated their results through the quantitation of *Ifna5* and *Ifnb* mRNA expression by quantitative RT-PCR on FACS-sorted populations of immune cells. Considering that neither of the approaches of these authors considered direct IFN-α protein recognition and that mice possessed 14 genes coding for IFN-α [91], DCs may not have been identified as major producers of IFN-I, given that they focused only on two of the genes responsible for IFN-α secretion, which could explain their results.

DCs are susceptible to hRSV infection [92,93,94,95]. However, DCs are poorly permissive to the virus as the infection is abortive and leads to poor production of new viral particles [54,93,96]. Human DCs subsets can be infected in different degrees by hRSV, as pDCs are less permissive than cDCs, and both cDC1 along with cDC2 are equally permissive to hRSV infection [97]. Interestingly, DC infection is enhanced by the presence of infected macrophages as in vitro models of airway epithelium have shown [98]. It can also occur through the internalization of antibody-coated hRSV through FcγRIIb and FcγRIII [96]. Moreover, FcγRIII knockout mice also show less severe hRSV symptoms, suggesting that this receptor contributes to hRSV pathogenesis, possibly by enhancing DC infection [96]. hRSV-infected DCs can promote airway obstruction, enhance disease, and promote more severe allergic responses in recipient mice [99].

**Figure 1:**
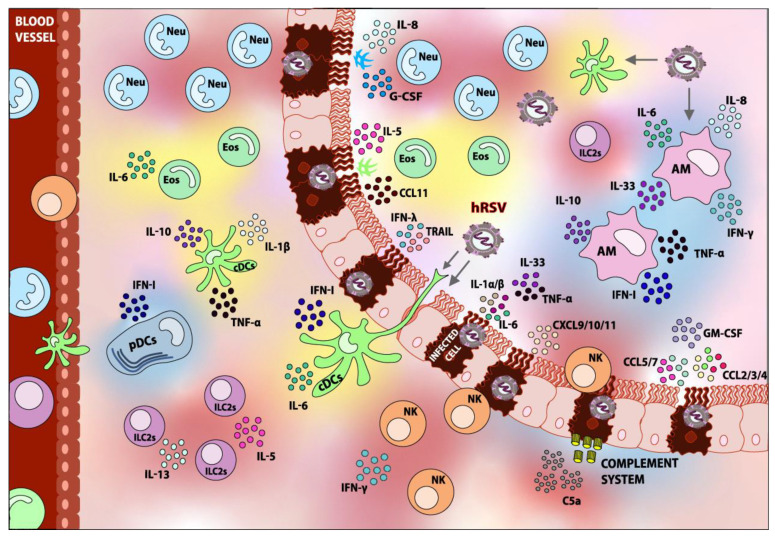
Innate immune response elicited upon hRSV infection. Following the detection of hRSV, various types of immune cells are recruited to the lung. Within the innate immune cells, Dendritic cells (DCs), Alveolar Macrophages (AMs), Neutrophils (Neu), Eosinophils (Eos), Natural Killer cells (NK), and Group 2 Innate Lymphoid Cells (ILC2s) can be found. Accordingly, the fixation and activation of the Complement System is detected. Most of these components play a role in the pathology of the infection. The role of the respiratory epithelium in the recruitment of innate immune cells can also be appreciated. Components shaded in blue are elements that contribute to hRSV disease-resolving, whereas components shaded in red are considered detrimental and contribute to hRSV pathogenesis overall. Elements depicted in yellow are those whose contributions to hRSV pathology are not well studied or are controversial. The arrows indicate the possible targets of infection.

hRSV infection induces DC maturation, as observed by the upregulation of markers, such as CD40, B7.1 (CD80), CD83, B7.2 (CD86), MHC-I, and MHC-II [54,94,100], although the upregulation of the latter is controversial. Moreover, the secretion of T_H_2-polarizing and inflammatory cytokines, including TNF-α, IL-1β, IL-6, and low quantities of IL-10 has been observed, as shown in Figure 1 [54,94,95]. The constitutive and TLR-induced secretion of IFN-I is impaired in infected DCs, but is still considered relevant [88,92,95,97,101,102,103]. Interestingly, the quantities and relative amounts of type I IFNs (α and β) and type III IFNs (λ) secreted by hRSV-infected DCs depend on the specific strain that infects the cell [102]. Finally, infected DCs exhibit a diminished capacity to activate CD4^+^ T cells [54,100]. Surprisingly, this phenomenon is not mediated by soluble factors—it is the expression of the nucleoprotein of hRSV on the surface of infected DCs which impairs the establishment of a proper immune synapse between both cell types [54,55].

During hRSV infection, poor or inadequate activation of T cells by DCs is not uncommon [54,100]. T_H_2 polarization is key for hRSV pathogenesis and is largely influenced by DCs. It has been shown that the cDC2 subset is capable of polarizing T cell responses in an IL-4Rα-dependent manner, which induces poor DC maturation and T_H_2 bias [104]. Moreover, the TLR3- and TLR7-mediated secretion of IL-33 by DCs could induce the generation of a T_H_2-biased adaptive response [105], and the secretion of TSLP by hRSV-infected epithelial cells can induce the maturation of cDCs, almost certainly to a T_H_2-polarizing profile [106].

As previously mentioned, cDCs are of myeloid origin and both subtypes are present in the lungs during hRSV infection [87], and in the BAL of hRSV-infected children [107]. Studies are controversial in determining their relative abundance in the peripheral blood of infected children [107,108]. However, it has been observed that the ratio between cDC1 and cDC2 is low in infected mice and could contribute to pathogenesis [88], since the cDC2 subset is more prone to polarize CD4^+^ T cells to a T_H_2 profile [104]. On the other hand, the cDC1 subset has vast antiviral capacities, given its high cross-presenting activity, which activates CD8^+^ T cells [109,110,111,112,113,114], further explaining why a low cDC1:cDC2 ratio is detrimental. Both cDC subtypes are equally susceptible to infection and are more permissive to hRSV than pDCs [94,97]. The infection with hRSV induces the upregulation of costimulatory markers B7.1 (CD80) and B7.2 (CD86) [94,97], and limits CD4^+^ T cell activation [88,94], as previously mentioned. Additionally, hRSV infection lowers IFN-α secretion in the cDC1 compartment and lowers IFN-β secretion in the cDC2 compartment [88,97].

On the other hand, pDCs are of mixed ontogeny and are characterized by their plasmacytoid appearance and vast secretion of IFN-I. These cells contribute greatly to disease resolution, lowering viral titers, and ameliorating pulmonary inflammation and airway obstruction [87,115,116].

Even though there is one report stating the opposite [90], the consensus is that pDCs are one of the foremost producers of type I interferons (IFN-I) in the lungs during hRSV infection [92,95,117]. Moreover, it has been observed that IFN-I secretion is TLR7/MyD88- and IFNαR1-dependent, but MAVS-independent, ruling out the involvement of RIG-like receptors (RLRs) in the triggering of IFN-I secretion [117]. Immunoglobulin-complexed hRSV (IC-hRSV) has been shown to induce TLR internalization and endosomal signaling in pDCs, which results in IFN-I secretion and can partly explain the observed TLR7/MyD88 dependency [118]. Additionally, the presence of IFN-I-producing infected monocytes or epithelial cells can further stimulate IFN-I secretion by pDCs, thus explaining the observed IFNαR1 dependency [118], without ruling out the possibility of autocrine IFN-I signaling.

Although pDCs do not interfere with CD4^+^ T cell responses [117,119], they do potentiate antigen-specific, IFN-γ-producing CD8^+^ T cell antiviral responses [116,117,119]. Despite their vast antiviral potential, hRSV-ALRTI children possess fewer pDCs in their peripheral blood than healthy controls [107,108], which also correlates with hRSV-induced asthma development in the future [120].

pDCs are susceptible to hRSV infection, albeit abortive and very scarce [97]. Infection readily induces the upregulation of CD40 and B7.2 (CD86) at even higher levels than cDCs [97]. Even though pDCs still produce IFN-I when infected [95], they do so at lower levels [88,101] and are less responsive to TLR-induced IFN-I secretion [88,92,95,103] and TLR-induced cytokine secretion [88].

Studies using neonate models of hRSV infection are extremely relevant, considering that newborns and children less than two years of age are an important high-risk population. Murine neonate models of infection have revealed that neonate pDCs are capable of processing and presenting antigens, but possess an intrinsic insufficient IFN-I response in comparison to mature adult pDCs [101]. Similar conclusions have recently been drawn for neonate cDCs [121]. Other studies regarding the role of cDCs in neonate models of infection reveal that neonate cDCs do not mature correctly in response to hRSV, since they do not upregulate costimulatory markers or CCR7, and maintain the expression of CCR5, which restrains migration to lymph nodes and retains DCs in the site of inflammation without proper maturation [122]. Another interesting observation is that the cDC2 compartment is less abundant in neonates, possibly leading to a weaker DC response against hRSV [110]. Interestingly, non-specific DC expansion in neonates leads to a better antiviral response based on the IFN-γ-secreting CD8^+^ T cell, which limits airway inflammation [116]. Furthermore, the cDC1 compartment is functionally limited in neonates when compared to adult cDC1s—neonate DCs internalize and process less antigen and upregulate B7.1 and B7.2 to a lesser extent [110].

Interestingly, lung migratory cDC1s from newborns establish different antigen-specific CD8^+^ T cell responses to adult cDC1s, giving rise to an immature-like immunodominant hierarchy among CD8^+^ T cells [110]. Briefly, these data suggest that there are differences between adults and neonates regarding the relative abundance of CD8^+^ T cell populations that recognize particular epitopes derived from hRSV antigens. The pattern of epitope dominance is relevant in the context of infection since it shapes the adaptive immune response and defines the epitopes from an antigen that will induce a more potent immune response [123,124,125]. Interestingly, neonate CD103^high^ cDC1s—but not CD103^low^ cDC1s—can establish the same immunodominant CD8^+^ T cell hierarchy that is observed in adult cDC1-mediated CD8^+^ T cell responses [111]. This suggests that CD103 upregulation in the cDC1 subset of neonate DCs is indicative of proper cDC1 development towards an adult-like phenotype. Lastly, the TLR4 and TLR9 stimulation of DCs in neonates leads to the cDC1 and cDC2 expansion and upregulation of B7.1 (CD80) and B7.2 (CD86), which also leads to an adult-like immunodominant hierarchy among CD8^+^ T cells [109].

To date, there are no clinical reports that describe the role of DCs in the pathology induced by the infection with hMPV [126]. However, in vitro studies, along with animal models, are able to demonstrate the role and function of DCs during this infection.

During the acute phase of infection with hMPV, DCs comprise the main immune cell population that senses this virus, along with macrophages and AECs [127,128]. As previously stated, DCs are susceptible to infection by hMPV, but the replication in these cells is not as efficient as in epithelial cells [75]. hMPV infection leads to an increase in the amounts of pDCs and cDCs in the lungs, reaching their peak at 8 and 10 d.p.i., respectively, and cDCs are found more abundantly and persist until 18 d.p.i. However, the CD103^+^ cDC1 subset decreases during the first weeks after the infection, but 8 weeks after the infection it reaches its normal amount [88]. The hMPV-infected DCs upregulate the maturation marker B7.1 (CD80) compared to non-infected cells, but additional markers, such as CD83 and B7.2 (CD86) do not exhibit significant differences. The infection is not able to cause the maturation of cDCs, but unlike hRSV, hMPV-infection has no cytopathic effects [129].

In vitro studies demonstrated that monocyte-derived DCs (moDCs) were capable of being infected by hMPV, but it was an unproductive infection [95]. The effect of the G and SH proteins during the infection with hMPV demonstrate a mechanism that hMPV might use to evade the immune system. Both of these proteins were not necessary to infect moDCs, and in their absence the rate of infection increased within moDCs [130]. The G and SH proteins of hMPV minimize the capability of internalizing the virus in moDC through a pathway similar to macropinocytosis, and by doing this, the virus decreases CD4^+^ T cell activation and, as a consequence, it reduces the T_H_1 response [130]. This correlates with previous findings, indicating that the infection of DCs with hMPV prevents the activation of naïve T cells [75,131].

**Figure 2:**
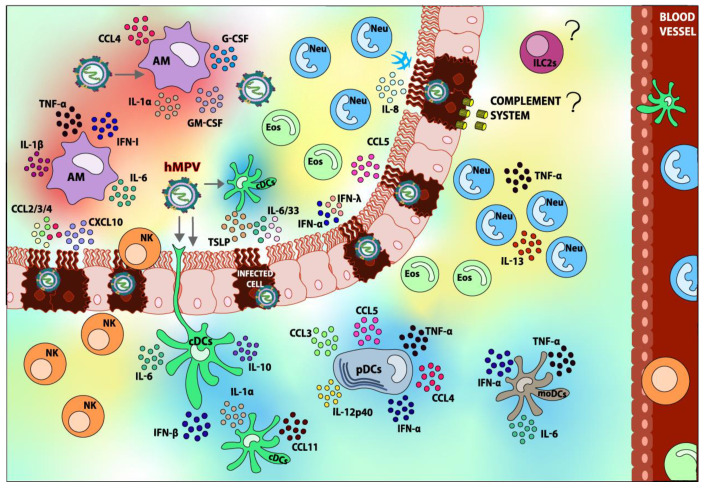
Innate immune response elicited upon hMPV infection. After the detection of hMPV, various types of immune cells are recruited to the lung. Within the innate immune cells, Dendritic cells (DCs), Alveolar Macrophages (AMs), Neutrophils (Neu), Eosinophils (Eos), and Natural Killer cells (NK) can be found. Most of these cells play a role in the pathology of the infection. However, Group 2 Innate Lymphoid Cells 2 (ILC2s) and the role of the Complement System during the infection have not been reported yet, and this lack of information is illustrated with “?” in the figure. The role of the respiratory epithelium in the recruitment of innate immune cells can also be appreciated. Components shaded in blue are elements that contribute to hMPV disease resolving, whereas components shaded in red are considered detrimental and contribute to hMPV pathogenesis overall. Elements shaded in yellow are those whose contributions to hMPV pathology are not well studied or are controversial. The arrows indicate the possible targets of infection.

When moDCs are infected with hMPV they can secrete low concentrations of IL-6 and TNF-α, and when these cytokines levels are compared to non-infected cells, the difference is not significant. However, moDCs secrete significantly higher IFN-α levels compared to non-infected cells [95]. On the other hand, hMPV-infected pDCs secrete the cytokines IFN-α, TNF-α, IL-12p40, CCL3, CCL4, and CCL5 upon infection, while in cDCs infected with hMPV, the cytokines IFN-β, IL-1α, IL-6, IL-10, and CCL11 (eotaxin) are secreted, as shown in Figure 2 [88]. IL-12p40 is a pro-inflammatory cytokine that is secreted in response to viral infection, and in the case of a hMPV infection, it plays an important role in the control of the inflammatory response and damage in the lung tissue. The absence of IL-12p40 increases the inflammation in the lungs and modifies the cytokine response, leading to deteriorated functionality of the lungs [132]. This observation suggests that the DCs play a protective role in the immune response against hMPV infection.

As mentioned before, pDCs are an important source of IFNs that are crucial for the appropriate antiviral response. A recent report demonstrated that the SH protein of hMPV suppresses the TLR7/MyD88 pathway in pDCs, leading to the inhibition of the IFNs expression [133]. The M2-2 protein of hMPV can impair the TLR7/9 pathway as well, thus inhibiting the secretion of IFN-I [134]. This indicates that SH and M2-2 proteins have a role in the evasion of the immune response, interfering with the proper response of pDCs.

IFNs are secreted via sensing the viral RNA. However, IFNs can be secreted through the sensing of IFN by the alpha interferon receptor (IFNAR) in the cells as well. The number of DCs decline when this receptor is absent during the infection with hMPV, causing the debilitated response of CD8^+^ T cell, since IFNAR enhances the increase in DCs in the first stage of replication [135].

In vitro studies have proven that the M protein can be released from the cell during the infection and can bind with DCs, internalizing this protein in a fast way. The M protein has the ability to induce the maturation of DCs and, as a consequence, the secretion of cytokines and chemokines, such as TNF, IL-1β, IL-6, IL-12p70, IL-8, and IL-10, that promote the inflammatory response [70]. Some reports have indicated that the infection with hMPV provokes low levels of IL-10, and that the low secretion of IL-10 might play a negative role in the severity of hMPV-infection within preterm infants [136,137].

In conclusion, DCs play varied roles during hRSV infection. Quite importantly, they are largely responsible for the T_H_2 polarization and poor CD4^+^ T cell responses against hRSV, especially when DCs themselves are infected. On the other hand, pDCs are an especially important and highly regulated source of IFN-I and are critical for the establishment of an antiviral state in the lung epithelium, as well as for the establishment of a CD8^+^ T cell-mediated antiviral response. Lastly, cDC1 responses also appear to be vital in the establishment of a proper CD8^+^ T cell response, whose immunodominant hierarchy is immature in neonates. As for DCs during hMPV-infection, it seems they play a protective role against the infection of this virus. The secretion of the cytokine IL-12p40 enhances the pro-inflammatory responses, and without it the inflammation causes intense damage in the lung tissue. The infection of DCs with hMPV impairs the proper priming of naïve T cells and can inhibit the signaling of TLR7/9, reducing the secretion of IFNs in pDCs. However, IFN levels can still be secreted by the different types of DCs. A comparison of the role of DCs between hRSV and hMPV infections can be found in Table 1.

### 3.2. Macrophages

Macrophages are within the first cells that protect the host against the viral infection, and there are two types in the lungs: the alveolar macrophages (AMs) and the interstitial macrophages (IMs) [138]. AMs are a type of macrophage, resident to the alveoli, that represent the first line of defense against respiratory pathogens [139]. Similarly to other tissue-resident macrophages, AMs are excellent phagocytes, possess a wide array of antimicrobial enzymes, can secrete ROS, can act as antigen-presenting cells, and mediate responses against both intracellular and extracellular pathogens [140]. They are also involved in the repairing of tissues after the infection has been resolved, through the secretion of anti-inflammatory cytokines as well as mitogenic factors [140].

In the case of hRSV, AMs are crucial for the early control of infection. During a hRSV infection, they represent one of the foremost and only sources of IFN-I, contributing to the establishment of an antiviral state in neighboring cells [90]. IFNs are secreted through RNA sensing by RLRs and their subsequent interaction with the mitochondrial antiviral signaling protein (MAVS) [90], although the possibility of TLR involvement should not be discarded, given that MyD88 is also necessary for proper IFN secretion [89].

Moreover, the depletion of AMs leads to a more severe disease caused by hRSV [141]. For example, it leads to airway obstruction [142], weight loss, and higher viral loads in the lungs [143] in murine models. Moreover, the activation of AMs with IFN-γ promotes the clearing of the viral infection [143]. Additionally, the expansion of the AM subset in mice recently recovered from allergically induced eosinophilia leads to a reduced hRSV immunopathology [144], supporting the notion that AMs play a protective role in hRSV infection.

The protective role of AMs can be at least partly explained through the previously mentioned secretion of type I IFNs, but also through the balanced and controlled secretion of pro-inflammatory cytokines, such as TNF-α, IL-6, and IL-8, upon the encounter with the live or inactivated virus, or upon AMs infected with hRSV, as shown in Figure 1 [141,145,146,147,148,149]. It is noteworthy that TNF-α has been shown to have antiviral properties in the context of their respiratory viruses, such as influenza [150]. Even though TNF-α is a pro-inflammatory cytokine in nature, which could enhance pulmonary inflammation and airway obstruction, the local and balanced secretion of TNF-α by AMs proves to be beneficial during hRSV infection, probably because of the promotion of an antiviral local state. Interestingly, AMs have been observed to promote the recruitment and activation of Natural Killer (NK) cells to the site of infection, further promoting a protective innate immune response [149].

AMs can be infected acutely by hRSV, as indicated above [151,152]. Interestingly, infection can occur through the internalization of IC-hRSV via the Fc receptors [153]. A report indicated that a persistently infected murine macrophage cell line for more than 87 passages can upregulate the expression of FcγRIIB and FcγRIII, which could render AMs more susceptible to hRSV infection [152]. Moreover, infected macrophages have shown to enhance DCs infection by hRSV, which can in turn lead to the development of a T_H_2 type adaptive immune response that is not efficient for virus clearance [98]. However, AM infection by hRSV is abortive and restricted, leading to the replication of only a few viral particles [145]. Interestingly, infection is also controlled in AMs lacking MAVS or IFNAR1, although virus and lysosome colocalization is less pronounced [145]. IL-6, TNF-α, IFN-α/β, and IFN-γ secretion is somewhat lower in infected AMs, but still relevant altogether [145,147,154]. On the other hand, phagocytosis and ROS production are also partially impaired in infected AMs [147]. Still, AMs are capable of playing a protective role, even when infected.

Nonetheless, some concerns have arisen regarding the possible role AMs could play in adaptive response polarization. Importantly, they can secrete IL-33 [105], a powerful driver of T_H_2 responses and harmful in the context of hRSV infection. IL-33 secretion was found to be induced by TLR7 agonists, suggesting a possible mechanism for IL-33 secretion during a hRSV infection [105]. Additionally, and even though a moderate amount of IL-6 promotes local inflammation and limits viral spreading, the uncontrolled secretion of IL-6 could result in harmful T_H_2 polarization [155].

Another controversial point is the finding that AMs can secrete considerable amounts of IL-10 at early time points of hRSV infection [156]. Concerns arise given the suppressive role that IL-10 can exert, which may result in the incomplete control of early infection.

In sharp contrast to hRSV infection, AMs seem to contribute to the pathogenesis caused by hMPV, rather than protect against the infection. In the bronchoalveolar lavage (BAL) of patients infected by hMPV, macrophages are found. Along this line, a biopsy of the lungs from these patients indicated chronic inflammation and the presence of alveolar macrophages in the tissue [157]. The characteristics of these macrophages found in the pulmonary tissue suggested the development of bronchiolitis in these patients.

During the acute phase of infection with hMPV, macrophages are among the main immune cell population to sense hMPV [127,128]. Studies performed in mice have demonstrated that macrophages reach the lungs 3 d.p.i., and are present until 7 d.p.i. [158]. The macrophages are recruited to the place of the infection due to the chemokine CCL2 (MCP-1), as a consequence of its high expression 1–5 d.p.i. This chemokine not only acts as a chemoattractant for macrophages but also has a role in the regulation of T_H_1/T_H_2 responses, especially since it promotes the secretion of IL-4, thereby stimulating the T_H_2 response even more [158].

AMs promote the spreading of hMPV though a macrophage infection-dependent mechanism, resulting in viral dissemination and the infection of the cells of the airway epithelium. Moreover, when AMs were depleted by the administration of a liposome-encapsulated clodronate suspension, hMPV infection caused a lesser decrease in the weights of infected mice, compared to non-depleted mice—the pathology score, along with the obstruction in the airways, decreased as well [141]. Additionally, the absence of AMs during the infection with hMPV leads to the decreased recruitment of neutrophils, but no significant difference in the recruitment of cDC2s [141]. The data indicate that AMs play an important role in the pathogenesis of the hMPV infection.

AMs are one of the main sources of IL-6, TNF-α, IFN-α/β, CCL4, GM-CSF, and G-CSF in the lungs, as shown in Figure 2. Furthermore, this study suggests that AMs are the main cells secreting IFN-I during a hMPV infection, similar to what happens during a hRSV infection [141]. A study performed in IFNAR^−/−^ mice demonstrated that AMs can function properly independently of IFNAR signaling, unlike IMs [135]. Additionally, the absence of this receptor does not cause AMs to be less recruited to the site of infection.

The detrimental role that AMs possess might be explained by the secretion of IL-1, since this cytokine is an important enhancer of inflammation in the lungs. The secretion of IL-1β is able to generate an inflammatory response by itself and promotes the secretion of more pro-inflammatory cytokines [159]. Moreover, it is known that the secretion of IL-1α/β can produce an inflammatory pathological response in the lungs in infectious contexts with other viruses, such as influenza, while playing no role in the clearance of the virus [160].

Generally speaking, the evidence points out that the activation of AMs in the lungs is a desirable response against hRSV. Their resilience against infection, their major contribution of type I IFNs, the secretion of adequate amounts of TNF-α and IL-6, and the recruitment of NK cells to the lungs are key for the early control of infection. Their possible role as T_H_2-polarizing agents would not be unexpected, but their involvement in the development of adaptive immune response seems to be minimal [149]. Hence, we highlight the positive role of AMs in the early control of hRSV infection in the lungs. On the other hand, the activation of AMs in the lungs does not seem to be an appropriate response against hMPV. The studies indicate that the presence of these cells does more damage than protection in response to this virus. It is worth mentioning that IL-1 has a pro-inflammatory effect that is linked with pulmonary damage. Moreover, these cytokines are not secreted by AMs during the hRSV infection, but they are during the hMPV infection. Additionally, studies comparing the amount of AMs recruited to the lungs by both viruses demonstrated that hRSV-infected mice are able to recruit more macrophages to the site of infection by day 7 p.i. compared to hMPV-infected mice [161]. The difference in cytokine secretion and the intensity of macrophage recruitment is where the difference between protection and damage might lie. A comparison of the role of AMs between hRSV and hMPV infections can be found in Table 2.

### 3.3. Neutrophils

Neutrophils are the most abundant type of immune cells in the bloodstream and possess important antimicrobial capacities. They are the first line of defense against many pathogens, given their notable phagocytic capacities, the release of proteolytic enzymes and other antimicrobial peptides via degranulation, and the formation of web-like structures specialized in the capture and elimination of pathogens, called neutrophil extracellular traps (NETs) [162].

During the infection with hRSV, the excessive infiltration of neutrophils and other granulocytes are a hallmark symptom of severe disease in children [163]. In vitro studies have shown that the secretion of IL-17 by T_H_17 CD4^+^ T cells along with the production of IL-8 (CXCL8) by epithelial alveolar cells are involved in the recruitment of neutrophils to the lungs, as shown in Figure 1 [164]. Moreover, hRSV infection and local IL-17A secretion have been shown to potentiate IL-8 secretion and enhance neutrophil recruitment [164,165,166].

Despite their antimicrobial capacities, a recent study shows that neutrophils are neither implicated in the elimination of hRSV in the lungs nor in the recruitment of effector or memory T cells to the lungs [167]. Moreover, the authors show that neutrophils do not contribute to disease severity since there was no pronounced weight loss or secretion of pro-inflammatory cytokines in the lungs (such as TNF-α, IL-1β, or IL-6) when the neutrophils were recruited to the lung via intranasal CXCL1 administration [167].

However, there is evidence that neutrophils can contribute to pathogenesis, causing airway inflammation and tissue damage. For instance, it has been shown that hRSV is able to induce NETosis in human neutrophils in vitro and in the BAL from children with hRSV-ALRTI [168,169], and that the F protein alone can induce NETosis in vitro through TLR4 activation [170]. Even though these NETs are able to trap hRSV and prevent further infection in vitro [168,169], their protective capacity in vivo needs to be evaluated. However, it has been observed that NET formation can contribute to airway obstruction in calves infected with bovine RSV (bRSV), a species closely related to human hRSV [169]. This would indicate that, although NETosis may contribute to limit viral replication, this occurs at the cost of developing one of the hallmark symptoms associated with ALRTIs—airway obstruction that might lead to bronchiolitis. Interestingly, the BALs of children with severe hRSV bronchiolitis contain neutrophils expressing the same levels of TLR4 protein on the cell surface as healthy controls but contain less TLR4 overall, which indicates that they possess lower levels of intracellular endosomal TLR4 [171]. Although this may not affect NETosis through the interaction of the cell surface TLR4 with the F protein of hRSV, it may influence the balance between the cell surface and endosomal TLR4 signaling upon activation. There may be a bias in signaling towards the activation of NF-κB and the subsequent secretion of pro-inflammatory cytokines, rather than the activation of IRF3 or IRF7. However, as already noted, Kirsebom and colleagues found no implication of neutrophils on the generation of the pro-inflammatory milieu observed in the lungs of infected mice [167].

Another harmful effect of neutrophils in the context of hRSV infection is their ability to release great amounts of reactive oxygen species (ROS) in a process called oxidative burst, non-specifically oxidizing biomolecules in the surroundings of the cell. Although quite effective for controlling pathogens, it can be very harmful to the tissues of the host. Interestingly, it has been observed that the pro-inflammatory milieu elicited by hRSV is capable of promoting an oxidative burst in neutrophils in vitro [172]. Moreover, hRSV is capable of downregulating the antioxidant enzymes needed for withstanding the oxidative stress, both in vitro and in the BALs of hRSV-infected children [173,174], leading to epithelial damage and lung injury.

In vitro studies have also shown that the hRSV infection of an epithelial alveolar cell monolayer promotes neutrophil transmigration and further supports the notion of excessive neutrophil recruitment to the site of infection [175]. More importantly, in a hRSV infection context, migrating neutrophils express higher levels of CD11b and myeloperoxidase, and induce considerable damage to the epithelial cell monolayer, as evaluated through monolayer integrity, cell count, and soluble LDH [175]. Additionally, it has been shown that the hRSV F protein is able to induce the neutrophil-mediated overexpression of mucin, one of the main components of mucus [176]. Thus, neutrophils may contribute to the production of excessive mucus that occurs in a hRSV infection and collapses the airways.

Regarding the infection with hMPV, during a study performed in infected patients aged between 1 and 16 years, numerous neutrophils were found in their BALF. Additionally, during a biopsy of the lungs from these patients, chronic inflammation in the airways was found [157]. Later on, it was shown that, in infants with hMPV-caused bronchiolitis, neutrophil infiltration during the infection could be detected [177].

During a study performed in hMPV-infected mice, it was shown that neutrophils represented the highest percentage of the immune cells evaluated that were present in the lung infiltrate, peaking neutrophilic infiltration 1 d.p.i. and decreasing over the course of infection. This extreme amount of neutrophils found in BAL and histological samples of bronchioli and alveolar spaces correlates with the damage caused in the lungs, such as interstitial pneumonitis and alveolitis [158,161]. Moreover, the presence of neutrophils can be observed up to 25 d.p.i. [178].

Reports have indicated that a high presence of neutrophils during other viral infections has been shown to induce acute damage in the lungs through NETs formation and the release of ROS, such as superoxide anions, as previously mentioned [179,180]. Moreover, neutrophils are considered of great importance regarding alveolar and bronchiolar inflammation, and in the damage caused by hMPV infection. During this infection, neutrophils are responsible for the secretion of TNF-α and IL-13, and/or for the stimulation of the secretion of these cytokines by other cell types, as shown in Figure 2 [128]. Interestingly, the secretion of TNF-α has the ability to recruit neutrophils to the site of infection as well [181].

IFN-I signaling plays a key role in the control of neutrophils, and when this signaling is absent, an increase in the recruitment of neutrophils is observed [182]. hMPV infection can interfere with this pathway by inhibiting the secretion of these cytokines, although low concentrations of IFN-α can still be detected [92]. The G protein of hMPV inhibits the secretion of IFN-I by interacting with RIG-I, thus inhibiting the RLR/MAVS-dependent activation pathway of NF-κB- and IRF3-regulated genes, which regulate the establishment of an antiviral cellular state [183]. Due to the inhibition of this pathway, along with the increase in cytokines, such as TNF-α, VEGF, IL-17, CCL3, CCL4, and CXCL2, in the presence of hMPV expressing the G protein, the authors concluded that this protein plays an important role in the recruitment of neutrophils to the alveolus [184,185].

Even though the presence of neutrophils seems to only contribute to the pathogenesis in the lung caused by the infection of hMPV, the lack of neutrophils has shown to aggravate the symptoms of the illness, increasing the inflammation in the lungs and making the recovery period even longer [179]. The lack of neutrophils causes an increase in the secretion of the cytokines TNF-α, IL-1β, IL-17, and 12p40, as well as the chemokines CXCL1, CXCL2, CXCL9, CCL2, and CCL11. Additionally, the absence of neutrophils does not seem to modify the presence of the virus in the lung, but does increase γδ T cell recruitment [179]. Thus, when neutrophils are not present during hMPV infection, the higher abundance of these T cells might mediate part of the increase in the lung damage.

Pediatric patients infected with either virus exhibited similar amounts of blood neutrophils [186]. However, one study observed a significantly higher amount of neutrophils in the BAL of hMPV-infected mice than in the BAL of hRSV-infected mice [161]. This might suggest that the number of neutrophils in the blood remains the same, but the number that infiltrates the lungs is different.

Even though there is controversial evidence regarding the role of neutrophils in the control of a hRSV infection, the sole presence of excess neutrophils in the airways may pose a problem for the disease severity. It is important to keep in mind that the excessive cellularity found in the BAL of hRSV-ALRTI almost certainly interferes with the normal physiology of lung function and may be an important obstacle for proper alveolar gas exchange, even when not considering the characteristic production of excessive mucus elicited by hRSV infection. As for hMPV, the role of recruitment neutrophils remains controversial since some reports indicate it causes damage in the alveoli and the bronchiole, but the lack of these immune cells greatly affects the management of the inflammation, enhancing the symptoms and, as consequence, increasing the amount of time the illness lasti. A comparison of the role of neutrophils during hRSV and hMPV infections can be found in Table 3.

### 3.4. Eosinophils

Eosinophils are granulocytes with important phagocytic capacities, usually overlooked when studying infectious diseases other than those that are helminth-related. These cells possess basic granules on their cytoplasm that contain four major classes of cationic proteins: the major basic proteins 1 and 2 (MBP-1/2), the eosinophil cationic protein (ECP), the eosinophil peroxidase (EPO), and the eosinophilic-derived neurotoxin (EDN) [187]. These proteins possess wide antimicrobial activities against not only parasites and helminths, but also against viruses, bacteria, and fungi as well [187].

EDN and ECP are of particular interest since they are RNases with antiviral properties against ssRNA viruses, particularly against pneumoviruses, such as hRSV [188]. It is also interesting to note that, although eosinophils express a somewhat limited array of TLRs, intracellular TLR7 is the most expressed of them all [189], further supporting the notion of a possible underappreciated role of eosinophils in the control of viral infections. These cells, however, are important effectors of T_H_2-based immunity and work synergistically with mast cells on many occasions [190]. Furthermore, they possess IgG and IgE receptors on their surface and are capable of secreting cytokines related with T_H_2 cell expansion, such as IL-2, IL-4, IL-5, IL-6, and IL-13, as well as various prostaglandins that are involved in asthma exacerbation [187,190,191,192]. Their location and migration to tissues are controlled by chemoattractant cytokines and chemokines, of which IL-5, IL-13, CCL5 (RANTES), and CCL11 (Eotaxin-1) are the most studied [193,194,195,196].

Eosinophilia is common in the BAL of children suffering from hRSV-ALRTI [197]. There is evidence that IL-5 and CCL11, both of which are powerful eosinophil chemoattractants, are produced in the lungs during hRSV infection [198,199,200]. Moreover, trials with a formalin-inactivated hRSV vaccine (FI-hRSV) were evaluated in the 1960s, which promoted vaccine enhanced disease (VED) and unfortunately resulted in two deceased children, showed that eosinophil counts in BAL from the deceased children were exceptionally high [201]. These facts lead to the thinking that the lung recruitment of eosinophils might not be the best-suited strategy to combat a hRSV infection. However, their role is controversial nowadays, as discussed below.

Firstly, there is evidence that eosinophils may be detrimental to resolving hRSV infection. For instance, it has been observed that eosinophils are susceptible to hRSV infection, which leads them to secrete higher amounts of IL-6, a T_H_2-polarizing cytokine, as shown in Figure 1 [202]. Moreover, eosinophils are capable of forming eosinophil extracellular traps (EETs), which are similar to NETs and are characterized by the catapulting of mitochondrial DNA in association with antimicrobial proteins [203]. EETs have been shown to occur during a hRSV infection of eosinophils in vitro [204] and probably promote airway obstruction, which could aggravate hRSV infection symptoms and may lead to the long-term development of asthma.

However, there is extensive evidence suggesting that eosinophils are beneficial in the control of hRSV infection and disease. One study found that the BAL of hRSV-ALRTI children with eosinophils expressing higher membrane CD11b (a marker of activated phagocytes) required less supplemental oxygen during the treatment [205]. On the other hand, there is evidence that there are elevated levels of EDN and other cationic proteins in nasal fluid and the other respiratory tract secretions of hRSV-ALRTI children [206,207,208], which may be beneficial considering their RNase and antiviral activity [188]. Lastly, many studies have reported that eosinophils are capable of capturing and inactivating hRSV—and other respiratory viral pathogens, such as influenza—and may contribute to the lowering of the viral load in the lungs of infected mice [189,209]. Considering that hRSV-ALRTI can induce the development of asthma in young children [210,211], it is interesting to note that asthma patients possess eosinophils with diminished virus-neutralizing capacities [209].

Lastly, some studies suggest that eosinophils may not play any role at all during hRSV infection, or at least during FI-hRSV VED. In a FI-hRSV immunized murine model, Knudson and colleagues show that eosinophils are not required for VED, and airway obstruction still occurs in mice lacking eosinophils—VED would be a consequence of T_H_2 polarization and not of eosinophilia, as previously thought [212]. However, studies by Su and colleagues show that IL-5- and CCL11-deficient FI-hRSV-immunized mice show no signs of airway obstruction or VED after a challenge with hRSV, suggesting that eosinophils do play a role in VED [213]. Thus, evidence regarding the role of eosinophils in VED is controversial and further studies must be performed to assert their importance in pathology.

Whether eosinophils are beneficial, detrimental, or just “bystanders” during a hRSV infection needs to be determined. Still, we believe eosinophilia must almost certainly be involved in the respiratory pathology of hRSV, because the cellularity of the infiltrate can interfere with the normal physiologic functions of the lungs (i.e., gas exchange), as discussed above for neutrophils. Moreover, the establishment of a signature T_H_2 adaptive immunity during hRSV infection leads to the secretion of antibodies, amongst whom IgE is almost exclusive of a T_H_2 profile. The secretion of IL-5 combined with IgE in the lungs might promote eosinophil migration and activation in the tissue because eosinophils possess IgE receptors. Activation leading to the degranulation of basic proteins could be harmful to the lung and may be implicated in the long-term development of asthma [208,214]. Although there is evidence of eosinophils playing a positive role in hRSV and other respiratory viral infection resolutions, their general implication in asthma development should not be overlooked, given their close and cooperative association with mast cells [190,191]. We believe further studies assessing the role of eosinophils in hRSV-induced asthma must be led to determine if eosinophils play a role in immunopathology in the long-term.

The role of eosinophils during hMPV infection is not clear, mainly because it has not been thoroughly studied. During a study performed in a patient infected with hMPV, the BAL was demonstrated to contain eosinophils while the patient was having a wheezing episode, shown in Figure 2 [157]. Another study was performed later with hMPV-infected pediatric patients, in which nasopharyngeal aspirates (NPA) were performed and the results showed a higher number of eosinophils compared to neutrophils. Most importantly, the presence of eosinophils cells was a parameter measured during the characterization of wheezing in patients infected with hMPV [186]. Since the hMPV infection leads to the recruitment of eosinophils to the lung, and these cells are associated with the development of wheezing and asthma, it is possible to suggest that eosinophils are the reason why these symptoms are present during a hMPV infection [215].

It was demonstrated that IRF7^−/−^ or IRF3/7^−/−^ mice developed eosinophilia during the infection with hMPV. Additionally, CCL24, a chemoattractant of eosinophils, also increased their concentration in the lungs [216]. Since hMPV can inhibit the homodimerization of IRF7, preventing the expression of IFNs, and the absence of this pathway recruits eosinophils, this virus promotes the recruitment of eosinophils as a consequence of inhibiting the secretion of IFNs [134,216].

Even though there was a presence of eosinophils in the samples from patients infected with hMPV, studies in BALB/c mice demonstrated that eosinophilia is not a common phenomenon in this model [217]. However, patients that have asthma are found to secrete IL-4, along with the generation of a T_H_2-polarized adaptive response. IL-4 is capable of promoting the differentiation and expansion of eosinophils that can cause a T_H_2 profile [218]. Based on this observation and the fact that the authors found levels of IL-4 in the hMPV-infected mice to be higher compared to the control, but significantly lower compared to hRSV-infected patients, they suggest that the levels of IL-4 were probably not enough to promote eosinophilia [217].

Eosinophils are more abundant in the blood of hRSV-infected patients than in the blood of hMPV-infected patients, and the same result is obtained when the samples are from NPA [186,219]. Thus, hRSV infection recruits a higher number of these cells, and this can cause symptoms such as wheezing and asthma [9,219]. The role of eosinophils during a hRSV infection is controversial, due to some studies suggesting that they have a protective antiviral role, while other studies suggest that these cells contribute to lung histopathology. On the other hand, the role that eosinophils have in the infection of hMPV is still unknown, and further studies are needed to reveal its role. A comparison of the roles of eosinophils between hRSV and hMPV infections can be found in Table 4.

### 3.5. Natural Killer Cells

Natural killer (NK) cells are innate lymphoid cells that mediate important antiviral responses, as well as those against intracellular bacteria and cancer cells. They have important cytotoxic capabilities mediated by perforin and granzyme, and are an important source of IFN-γ [220].

Studies regarding the presence of NK cells in the BAL of hRSV-infected children are controversial. Some studies have found a high presence of these cells [107,221], whereas others have reported a lower proportion of them in contrast to control samples [222,223]. Interestingly, one of the later studies found that the few NK cells detected were highly activated, suggesting a possible involvement of this cell type during hRSV infection [223]. In murine models, NK cells are recruited to the lung early in the infection, near 4 d.p.i. [224,225,226]. Moreover, the presence of NK cells as late as 21 d.p.i. has also been reported [227]. Even though CCL3 is a chemokine known to be a powerful chemoattractant for both NK cells and CD8^+^ T cells, its concentration in the BAL samples from the hRSV-infected mice is so low that it does not induce NK cell chemotaxis [228]. However, it has been reported that the supernatant of the hRSV-exposed macrophages does induce NK cell chemotaxis [228], suggesting a possible mechanism by which NK cells migrate to the lungs during hRSV infection.

It has been observed that lung NK cells at early time-points of infection express activation markers and secrete high quantities of IFN-γ [224,226,229,230,231]. Although IFN-γ is a hallmark antiviral cytokine, its early secretion is associated with pulmonary inflammation and airway obstruction during the hRSV infection [227,231]. Moreover, an excess of NK cell-derived IFN-γ has also been recently found to inhibit the development of humoral adaptive responses, leading to a complete lack of hRSV-specific antibodies in neonates, as shown in Figure 1 [229]. NK cells have been shown to be protective in CD8^+^ T cell-depleted murine models, in which IFN-γ secretion is impaired and NK-cell derived IFN-γ is not excessive since there is no CD8^+^ T cell-derived IFN-γ [225].

NK cell activation and IFN-γ secretion have controversial antiviral activities. Even though NK cell activation is associated with lower viral loads [232], and their cell depletion is associated with higher viral loads [231], less severe respiratory symptoms [231], the generation of an inadequate T_H_2 profile [230], lower levels of pulmonary CD8^+^ T cells [224], and eosinophilia [224]. Some studies have found that NK cell depletion does not affect eosinophilia, neutrophil infiltration, or viral load [226]. Thus, although there are reports that highlight their antiviral activity, they worsen the symptoms of hRSV disease and can hardly be considered protective.

The hRSV infection of both neonate and adult NK cells has recently been reported [233]. NK cells are poorly susceptible to hRSV infection, but the Ig-coating of hRSV greatly enhances the infection [233], suggesting yet again a possible role of the Fc receptors in the hRSV infection of immune cells. Moreover, hRSV-infected NK cells secrete higher amounts of IFN-γ than non-infected NK cells, while perforin secretion remains unaltered [233]. Thus, the hRSV infection of NK cells possibly leads to a worsening of the respiratory symptoms caused by hRSV through IFN-γ-mediated pulmonary inflammation.

Unlike hRSV, the information concerning NK cells and their role in the infection with hMPV is rare, and most reports do not agree with one another after an infection with hMPV, the number of NK cells increases up to 3 d.p.i. in the lungs, and they are completely functional, as shown in Figure 2 [234]. However, the protective role of the NK cells against the hMPV-infection is controversial, since some studies suggest that the lack of these cells does not affect the viral load, while others indicate that the viral load increases in their absence [81,234]. In the latter study, it was found that the viral load of hMPV peaked at 28 d.p.i. and was still detectable until 60 d.p.i. [234]. Even though these results are different, it has to be considered that the authors used different approaches to achieve the depletion of NK cells in order to study their functions.

In the study where the depletion of NK cells did not change the viral load, an anti-NK1.1 antibody was used to accomplish the depletion of these cells. This antibody can produce the depletion of both the NK cells and natural killer T (NKT) cells, and this factor might affect the results [234,235]. In this study, it was found that NK cells do not interfere with the secretion of cytokines IL-2, IL-4, or IFN-γ, but do increase the expression of IL-10 compared to the non-depleted mice [234]. Additionally, the absence of NK cells does not contribute to the damage associated with the immune response against the hMPV infection. Reports indicate that NK cells are able to regulate macrophage activation, but the lack of NK cells does not affect the number of macrophages in the lungs during the infection with hMPV [220,234]. Moreover, CD4^+^ T cells and CD8^+^ T cells in the lungs do not vary either. According to the authors, NK cells are not essential for the clearance of the virus since their absence does not make any difference in the recruitment of immune cells [234].

NK cells have receptors that can inhibit or activate their state of activity, and within these receptors, the natural cytotoxicity receptors (NCRs) can be found, which are one of the classes of receptors with the most influence on the activation of NK cells. NKp46 is one of these receptors in humans, while NCR1 is its homologue in mice [236]. These receptors are activated after the infection with hMPV by an unknown ligand that is not a viral protein [237]. Moreover, NCR1 is important for the NK cell-mediated control of hMPV infection in the murine model since its absence is associated with an increased viral load [237].

Interestingly, there is a differential recruitment of NK cells during the infection with hRSV or hMPV. One study demonstrated that NK cells were significantly more recruited to the site of infection under hMPV than hRSV by day 4 p.i.; however, by day 7 p.i., the amounts of NK cells were similar [161].

In conclusion, NK cells play a detrimental role during hRSV infection, promoting an inflammatory milieu that contributes to lung inflammation and promotes airway obstruction, thus contributing to the pathology associated with the disease, despite the possible contribution of NK cells to viral clearance. On the other hand, NK cells do not seem to enhance the immunopathology caused by hMPV and their protective or pathogenic capacities remain to be elucidated, although there is some evidence that suggests that NK cells contribute to viral clearance. Even though it seems that both viruses promote different NK cell responses, it is clear that the recruitment of these cells to the lungs takes place. Further insights regarding their specific roles during pneumovirus infection are needed to further comprehend the immunopathology caused by these viruses. A comparison of the roles of NK cells during hRSV and hMPV infections can be found in Table 5.

### 3.6. Innate Lymphoid Cells

Innate lymphoid cells (ILCs) are cellular components of the innate immune system of lymphoid origin, which were discovered nearly a decade ago. They stem from the common lymphoid progenitor (CLP), further mature into different subsets, and migrate to mucosal tissues, poised in key positions for pathogen recognition and subsequent rapid activation [238].

Interestingly, NK cells represent the one and only cytotoxic class of ILCs known to date and are related to other ILCs only early in development [238]. These cells have already been discussed above. The other classes of ILCs are all non-cytotoxic and include lymphoid tissue inducing (LTi) cells, ILC1s, ILC2s, and ILC3s [238]. LTis are responsible for the generation of lymphoid tissues, such as lymph nodes and other secondary lymphoid organs, during embryonic development [238]. ILC1s are able to secrete IFN-γ and TNF upon activation, thereby controlling infections by intracellular bacteria and viruses, and they can express T-bet, the T_H_1 hallmark transcription factor [238]. ILC2s secrete various T_H_2-related cytokines upon activation, such as IL-4, IL-5, IL-9, IL-13, IL-25, and IL-33, and express GATA3, a T_H_2 hallmark transcription factor [238]. Interestingly, these cells are of great importance in the immune responses against helminths and other parasites, as well as in the tissue-repairing and allergic airway responses [239,240,241,242,243]. Finally, ILC3s can secrete IL-17A, IL17-F, and IL-22 upon activation, which are relevant for the clearing of extracellular bacteria through the induction of the secretion of antimicrobial peptides on epithelial cells, and are characterized by RORγt expression, similar to T_H_17-polarized CD4^+^ T cells [238].

It is important to note that, although there are similarities between ILC subtypes and T_H_ profiles, ILCs do not possess TCRs, do not exhibit wide genetic diversity, and are not antigen-specific [238]. However, they have been shown to interact with CD4^+^ T cells through antigen presentation in class II MHC, which activates both the ILC and the CD4^+^ T cells in an IL-2-dependent fashion. This is especially true for ILC2s, which have been shown to strongly promote the T_H_2 responses [238,241].

There are few reports regarding the role of ILCs during the hRSV infection, especially in humans. However, it has been observed that elevated levels of ILC2s in the BAL of the hRSV-infected children correlate with a higher disease severity [244], probably through the generation of a stronger detrimental T_H_2 response.

Studies using murine models have demonstrated that a significant expansion of the ILC2 subset occurs in the lung early in hRSV infection [245,246,247,248]. Moreover, these ILC2s are IL-13-producing, as shown in Figure 1 [246], and could thus skew the adaptive response towards a T_H_2 response. It has been suggested that TSLP is responsible for the activation and expansion of this IL-13-producing subset, since TSLPR KO mice exhibited diminished weight loss, lower levels of IL-13-producing ILC2s, lower lung IL-13 levels, and lower airway hyperresponsiveness (AHR) compared to WT mice [246]. TSLP neutralization also lowered the IL-13 levels in the lungs of infected WT mice [246]. Interestingly, IL-33, a potent driver of T_H_2 responses which is upregulated early in hRSV infection, is not involved in ILC2 expansion in the lung [246], but can enhance IL-13 secretion by these cells [245].

It has been reported that uric acid is produced during hRSV infection and can also promote hRSV pathogenesis by enhancing TSLP and IL-33 production [247], which leads to ILC2 accumulation and ultimately drives T_H_2 responses. Xanthine oxidase is the enzyme responsible for the degradation of nucleic acids and contributes to uric acid production in the lungs by this pathway, which is known to induce allergic responses [249]. Targeting xanthine oxidase with inhibitors during hRSV infection has been shown to reduce TSLP and IL-33 levels in the lung, as well as ILC2 accumulation [247]. Targeting IL-1R with antagonists can also reduce ILC2 accumulation in the lung [247].

Interestingly, ILC2 activation appears to be dependent on interaction with CD4^+^ T cells during hRSV infection, which allows for ILC2 expansion and activation—as measured by IL-5 and IL-13 production—in an IL-2-dependent manner [248]. ILC2s from the hRSV infected mice could also be activated by IL-2 treatment, further supporting the notion that CD4^+^ T cell-derived IL-2 is what activates ILC2s [248]. On the other hand, the adoptive transfer of pulmonary ILC2s from the hRSV-infected mice enhances the IL-4-, IL-5- and IL-13-expressing CD4^+^ T cell expansion, but not the IFN-γ-expressing CD4^+^ T cell expansion, in infected mice [250]. Moreover, ILC2-mediated CD4^+^ T cell activation requires cell–cell contact and OX40–OX40L interaction [250].

Finally, it has been observed that the expression of the transcription factor STAT1 is critical for a proper ILC response against hRSV. It has been observed that STAT1 KO mice exhibit a lower recruitment of IFN-γ-producing ILC1s during hRSV infection, as well as higher numbers of IL-5- and IL-13-producing ILC2s, and IL-17A-producing ILC3s, which are detrimental to the hRSV infection since they promote harmful T_H_2 and T_H_17 responses [251]. This further supports the importance of STAT1 during the hRSV infection, given its participation in the antiviral type I, II, and III IFN responses.

On the other hand, there are no studies regarding these cells and their relationship with hMPV infection. It would be of interest to investigate whether these cells are affected by the infection of hMPV and if they contribute to lung damage.

Overall, the data suggest that the antigen presentation on MHC-II by ILC2s could be the mechanism by which ILC2s induce CD4^+^ T cell activation and subsequent IL-2-dependent ILC2 activation, as well as T_H_2 polarization on CD4^+^ T cells during hRSV infection. This would make sense, given that ILCs have been observed presenting antigens on MHC-II, albeit less efficiently than DCs [241,252]. However, the capacity of ILC2s of presenting hRSV antigens on MHC-II during a hRSV infection remains to be determined. Nonetheless, it is clear that ILC2s contribute to hRSV pathogenesis through the secretion of IL-5 and IL-13, and promote detrimental T_H_2 responses, both of which can ultimately lead to distinctive hRSV-caused eosinophilia and immunopathology.

ILC1s could possess antiviral capacities and be beneficial for hRSV infection, but studies regarding this cell type have not been performed. Similarly, studies regarding the role of ILC3s should also be performed to establish the precise role that these cells play during hRSV infection. A summary of the role of ILCs in hRSV infection can be found in Table 6.

### 3.7. Complement System

The complement system is a group of soluble plasmatic proteins with intrinsic and sequential protease activities that possess cytolytic and proinflammatory properties. The cascading of protease activities culminate in the formation of a membrane attack complex (MAC) on the surface of the target cell, inducing the formation of a large pore on the cell membrane that leads to the loss of osmotic control and lysis [253]. Additionally, the proteolytic cleavage of several components of the complement system leads to the liberation of anaphylatoxins, such as C3a and C5a, that enhance vascular permeability and are powerful chemoattractants of immune cells [253]. It is noteworthy that complement fixation and activation can occur through multiple pathways. Complement fixation is mediated by antibodies in the classical pathway, whereas in the lectin and alternative pathways, it is antibody-independent. Moreover, the lectin pathway relies on the initial lectin-mediated fixation of the complement [253].

Initial observations during the 1980s describe complement fixation on the surface of the hRSV-infected cells in vitro, through both the classical and alternative pathways as shown in Figure 1 [254]. Interestingly, both syncytial and non-syncytial infected cells were able to bind complement proteins, although fixation through the alternative pathway was higher in syncytial than in non-syncytial infected cells [254]. Evidence of complement fixation on epithelial cells from nasal swabs of the hRSV-infected children came shortly thereafter [255]. Complement fixation was observed occurring shortly after the onset of the symptoms, and complement proteins were almost completely bound to IgA, IgG and IgM, indicating the preponderance of the classical pathway of the complement during the hRSV infection [255]. Even though early studies have found complement proteins to induce cytolysis of the hRSV-infected cells on their own, it has been observed that cell lysis can be enhanced in the presence of neutrophils during the hRSV infection [256].

More recently, it has been observed that two anti-G hRSV neutralizing antibodies (CB002.5 and CB017.5) require the presence of the complement to neutralize hRSV infection in Vero cells [257]. It has also been shown that C5-deficient mice and cobra venom factor (CVF)-treated mice (which are complement-depleted) are more susceptible to hRSV infection when treated with 18A2B2, a prophylactic and protective anti-G antibody [258]. Lastly, it was observed that CVF-treated complement-depleted mice that are exposed to hRSV do not exhibit higher or lower viral loads in the lungs, compared to non-depleted mice [259]. However, complement depletion completely abolishes the protective response observed when anti-hRSV antiserum is administered [259], further indicating the relevance of the classical pathway of the complement system in protective antibody-mediated responses against hRSV.

However, the complement system also plays a pathogenic role during hRSV infection, mediated mainly by the anaphylatoxins C3a and C5a produced during the proteolytic cascade of complement proteins. For instance, hRSV-infected mice lacking the receptor for C3a (C3aR1) exhibit lower AHR, lower neutrophil infiltration, higher macrophage recruitment to the lung, and lower viral load than WT mice [260]. Moreover, they possess lower quantities of T_H_17-related cytokines in the lung (such as IL-1β, IL-6, IL-17A, and IL-21), as well as lower transcription levels of RORγt, without changes in TNF-α, IFN-γ, IL-4, or IL-5 levels [260]. These data suggest that C3aR1 regulates the generation of pathogenic neutrophil-recruiting T_H_17 responses during hRSV infection [260]. On the other hand, another study shows that treatment with C5aRA—an antagonist of the C5a receptor—leads to lower pulmonary damage, lower AHR and lower infiltration of neutrophils in the lung and BAL, as well as a reduced viral load in the lung during hRSV infection [261]. Moreover, AHR and the infiltration of inflammatory cells are also reduced in asthma murine models during hRSV infection [261]. These data suggest that the activation of C5aR is also pathogenic and enhances AHR and pulmonary infiltration during hRSV infection and is possibly implicated in the control of asthmatic responses [261]. This is especially important, considering that C5a levels are increased in lung and BAL, and C5aR expression is upregulated in airway epithelial cells during hRSV infection [261].

Consistent with its involvement in antibody-mediated immune responses, the complement system has also been proposed to play a role in FI-hRSV VED, since there is a significantly higher deposition of C3 in FI-hRSV-immunized mice during hRSV infection, as well as C4 deposition in the lungs of both patients who died during the FI-hRSV vaccine trial [262]. Surprisingly, FI-hRSV-immunized C3-deficient mice exhibit much lower AHR than FI-hRSV immunized WT mice during hRSV infection [262]. It is likely that complement-mediated AHR is caused by the uncontrolled production of anaphylatoxins, C3a and C5a, given their relevance in mediating the lung infiltration of immune cells.

Considering that part of hRSV-induced pathology is mediated by anaphylatoxins, it is interesting to note that hRSV has been shown to induce the expression of CD59 in vitro in A549 cells [263]. CD59 is an inhibitor of the MAC formation, and inhibits the polymerization of C9 in the cell surface, thus inhibiting the final step in the formation of the membrane pore [264,265,266]. Hence, hRSV possibly inhibits the complement-dependent lysis of the infected epithelial cell, without preventing anaphylatoxin production and subsequent cellular infiltration and increased AHR. Consequently, the combined activity of MAC inhibition and anaphylatoxin secretion possibly contributes to increase the pathology and poor viral clearance.

On the other hand, there are no studies regarding the fixation and activation of the complement system in a hMPV infection. Similarly, there are no studies regarding the role of complement-derived anaphylatoxins or the role of the complement proteins in humoral or cellular responses against hMPV. Certainly, this is an interesting and unexplored area that might provide insight into the components that mediate cellular infiltration and antibody-based responses in a hMPV infection.

In conclusion, the classical pathway of the complement system mediates part of the antibody responses against hRSV and is critical for a proper humoral response. The complement system possibly promotes viral clearance through the lysis of infected cells through both classical and alternative pathways. However, the excessive or uncontrolled production of anaphylatoxins can be detrimental during hRSV infection and can promote cellular infiltration and increased AHR, as well as inefficient viral clearance. A summary of the role of the complement system in hRSV infection can be found in Table 7.

## 4. Role of the Respiratory Epithelium during the Immune Response

The respiratory epithelium is an important driver of the immune response against respiratory viruses, as these cells are implicated in the secretion of large quantities of chemokines upon infection. This will attract immune cells to the site of infection, as well as other various cytokines that modulate the immune response [267].

For hRSV, epithelial cells have been seen to contribute to the promotion of local inflammation and angiogenesis, the recruitment of various inflammatory cell types, and the development of a harmful T_H_2 adaptive immune response. Many of these studies are based on in vitro models, and we only discuss the secretion of cytokines that have been observed in at least one type of human primary cell culture [118,230,247,267,268,269,270,271,272,273,274,275,276,277,278,279,280,281,282,283,284,285,286,287,288,289,290,291,292,293,294,295,296,297,298].

Regarding acute local inflammation, the hRSV-infected epithelial cells have been shown to secrete IL-1α/β, IL-6, HMGB1, TNF-α, VEGF, and FGF [118,268,269,270,271,272,273,274,275]. These cytokines are implicated in the generation of local and generalized inflammation and contribute to angiogenesis, endothelial cell activation, vasodilation, immune cell transvasation and activation, and pyrogenesis. Importantly, the cytokines IL-1α/β are systemic pro-inflammatory mediators that can contribute to a self-perpetuating inflammatory response, both locally and systemically. During a hRSV infection, all of these cytokines are produced as early as 24 h.p.i. [268,269], and some are secreted at even earlier time points, such as pro-inflammatory IL-1α (12 h.p.i.) [276], endothelium-activating VEGF (6 h.p.i.) [270] and IL-6 (6 h.p.i.) [273].

Epithelial cells are deeply implicated in the recruitment of many immune cell types, among which neutrophils, monocytes, eosinophils, and NK cells are found. The extensive secretion of neutrophil chemoattractant CXCL8 (IL-8) has been found to occur during the infection with hRSV as early as 2 h.p.i. and to be steadily secreted for as long as 6 d.p.i. [165,272,273,276]. Moreover, G-CSF secretion during the first 2 days of infection [268,269,275] probably promotes neutrophil survival and stimulation in the lungs. The hRSV infection can also secrete monocyte chemoattractants, such as CCL3 (MIP-1α), CCL4 (MIP-1β), and CCL7 (MCP-3), which are secreted 24 h.p.i., while CCL2 (MCP-1) is secreted 6 h.p.i. [269,275,276,277,278] and CCL5 (RANTES) can be consistently detected between 12 h.p.i. and 6 d.p.i. [269,272,274,279,280,281]. Epithelial cells also secrete GM-CSF upon the infection with hRSV [269,282], which can promote monocyte survival and differentiation. After early neutrophil (2 h.p.i.) and monocyte (6 h.p.i.) chemoattractants secretion, eosinophil chemoattractants—CCL5 (RANTES) and CCL11 (eotaxin)—secretion follows while the hRSV infection continues, the latter occurring around 12 h.p.i. [269]. Finally, NK cell chemoattractants CXCL10 (IP-10), CXCL9 (MIG), and CXCL11 (IP-9) are secreted at later time points, near 18, 24, and 48 h.p.i., respectively [269,274,275,276,283]. Even though NK cells are also recruited via CCL3/4/5 [267], the secretion of CXCL9/10/11 probably boosts recruitment in the later stages of infection, approximately 1 to 2 d.p.i. Antiviral molecules, such as TRAIL and IFN-λ have also been shown to be secreted due to the infection with hRSV around this time, and almost certainly contribute to the restriction of viral replication in conjunction with NK cells [269,274,279,284]. A detailed review of cytokine secretion containing a model of the timeline of the secretion of chemoattractants by airway epithelial cells has been recently published [267].

Finally, hRSV infected epithelial cells have been shown to secrete T_H_2-polarizing cytokines by 24 h.p.i., such as TSLP, CCL2 (MCP-1), MCP, HMGB-1, IL-25, and IL-33 [230,247,268,269,285,286,287]. These cytokines greatly influence antigen-presenting cells and ILC2s, driving IL-4, IL-5, and IL-13 secretion that will ultimately lead to antibody-mediated adaptive immune responses, rather than a cytotoxic response [288]. Moreover, epithelial cells have been found to interact with CD4^+^ T cells via the Jagged/Notch-1 contact-dependent axis, directly influencing T_H_2 differentiation in vitro [289].

Regarding the hMPV infection of AECs, it seems to induce a T_H_2 response against infection due to the secretion of TSLP and IL-33 [290]. TSLP is a cytokine found to be elevated during asthma, contributing to the pathogenesis of the infection, and promoting a T_H_2 cytokine response through DCs [291,299]. Even more, it has been suggested that the activation of this cytokine pathway plays an important role in the pathogenesis caused by hMPV [128]. As for IL-33, it promotes the secretion of cytokines from a T_H_2 response and it is known to contribute to the damage produced in the lungs [292].

The hMPV-infection of epithelial cells from the lung causes the secretion of the chemokines IL-8 (CXCL8) and CCL5 (RANTES). IL-8 can be detected as early as 6 h.p.i. while CCL5 can be observed after 12 h.p.i., although both cytokines can achieve the highest secretion at 48 h.p.i. [293]. The secretion of IL-8 is associated with the recruitment of neutrophils to the site of infection, and its significant secretion has been associated with bronchiolitis caused by hMPV infection [158,293]. CCL5 secretion has been involved in the recruitment of both neutrophils and eosinophils, as well as being related to the increase in asthmatic symptoms in the lungs [294].

Cytokines IL-6, CXCL10 (IP-10) and CCL2 (MCP-1) were also found to be secreted by epithelial cells infected by hMPV. IL-6 reaches the highest concentration 48 h.p.i., while CXCL10 and CCL2 reach their peaks 24 h.p.i decreasing their levels at 48 h.p.i. Even though CCL3 (MIP-1α) and CCL4 (MIP-1β) secretions are higher compared to non-infected controls, their levels remain low [293]. It has been shown that the increase in CCL5 in the lung can stimulate the secretion of CXCL10, CXCL2 (MIP-2), and CCL2 [294]. IL-6 can cause general inflammation in the lungs, while CXCL10 induces the recruitment of T cells and NK cells, while CCL2 along with CCL3 and CCL4 promote the recruitment of monocytes, macrophages and NK cells [295,296]. Additionally, the respiratory epithelium upon the infection with hMPV can secrete IL-1α, IL-11, and IL-32 [300]. IL-1α is involved in the vasoconstriction and activates the epithelium, and IL-32 stimulates the secretion of other cytokines, such as TNF-α and IL.6, contributing to the inflammatory response [301,302]. To date, no evidence has demonstrated the secretion of colony-stimulating factors in the respiratory epithelium infected with hMPV.

Lastly, the infection with hMPV is able to induce the secretion of IFN-α/β, and this secretion can activate IFN-signaling from other epithelial pulmonary cells. However, the amounts of epithelial cell-derived IFN-α are lower compared to those derived from moDCs [293]. Additionally, hMPV infection causes an increased type III IFNs expression in AECs, such as IL-28 and IL-29. These cytokines have been associated with a protective role against hMPV in the lungs [297,298].

The immunopathology associated with hRSV infection is largely mediated by the secretion of inflammatory cytokines and various chemokines that lead to neutrophilia and eosinophilia, both hallmark symptoms of the hRSV infection, and the secretion of T_H_2-polarizing cytokines, which overall lead to a poor response against hRSV. As for the hMPV-infection, it seems that most of the cytokines secreted by AECs promote a T_H_2 inflammatory response, contributing to the obstruction and damage of the tissue. However, the expression of IFN-λ contributes to the protection of the pulmonary epithelium. A comparison between cytokine secretions from the RSV- and hMPV-infected epithelial cells can be found in Table 8.

## 5. Prophylactic and Vaccine Approaches against These Respiratory Viruses

Vaccines are a great—if not the best—way of preventing virus-caused illness. However, neither hRSV nor hMPV have approved vaccines yet [303]. Many prototypes are currently being studied. Another relevant and effective prophylactic approach against these viruses is the use of monoclonal antibodies (mAbs). Both of these treatments are discussed below.

In the case of hRSV, there are a few monoclonal antibodies and several vaccine candidates with prophylactic potential. These are shown in Table 9 and are briefly discussed below.

Palivizumab is a F-hRSV-targeting monoclonal antibody that is currently on the market and represents the main prophylactic approach against hRSV. It has been shown to be protective and to effectively neutralize hRSV [304,305,306,307]. However, its high cost and the necessity for multiple dosage for exerting protection are the main limitations palivizumab possesses as a prophylactic strategy [305,308,309].

Motavizumab is an optimized variation of palivizumab, obtained by fine-tuning the amino acids present in the complementarity determining region of the antibody [310]. Although it has been shown to be more neutralizing and protective than palivizumab, cutaneous adverse effects have been observed in infants that were treated with motavizumab [311,312]. Hence, the FDA did not approve of its use in humans.

Other strategies involving monoclonal antibodies are MEDI8897, a F-targeting antibody currently ongoing a phase 3 clinical trial [313,314], and a novel anti-N targeting monoclonal antibody, currently being developed and tested in preclinical models, as shown on the PATH website. The history of antibody-based prophylaxis for hRSV can be reviewed in [309].

Regarding vaccination strategies against hRSV, there are many candidates currently ongoing clinical trials [16]. However, no safe and effective vaccine against hRSV has been approved yet. Current candidates in ongoing clinical trials include live-attenuated, chimeric, vector-based, particle-based, and subunit vaccines. No DNA vaccines or whole-inactivated vaccines are currently ongoing clinical trials [16]. A thorough and comprehensive review of these strategies can be found in [315].

Many live-attenuated vaccines are currently ongoing phase 1 clinical trials. These vaccines consist of deletion-bearing hRSV strains [315]. The most common antigen that is deleted is the M2-2 protein, which is involved in the control of transcription and replication processes. One of these vaccines was shown to be over-attenuated and therefore did not pass phase 1 clinical trials [316]. Other approaches involve the deletion of NS2, a non-structural protein responsible for the inhibition of IFN production in infected cells [317,318].

A chimeric BCG-vectored vaccine expressing the N protein of hRSV was recently completed in a phase I clinical trial (data not shown). The rationale behind this formulation is supported by the fact that the N protein is expressed on the membranes of infected cells, thus exposing this antigen [54,55]. Interestingly, it was shown to induce a protective humoral response and a balanced T_H_1 response [319,320]. Another main advantage of this vaccine candidate is its proposed dual capacity of inducing immunity against both hRSV and *Mycobacterium tuberculosis* in immunized newborns.

Vector-based vaccines have also been developed and are currently ongoing phase 2 clinical trials. Three of these vaccine candidates are based on the adenovirus-mediated delivery of antigens and one of them is based on a modified vaccinia Ankara virus. All are in ongoing phase 2 clinical trials. These vaccines rely on poorly replicating vectors and induce protective humoral and cellular immunity against hRSV antigens—the F protein in particular [315].

Particle vaccine candidates are being developed and are in ongoing clinical trials for three target populations: pediatric, elderly, and maternal. They are based on the delivery of stabilized post-F antigens in the form of nanoparticles, and are among the most promising candidates [315].

Finally, there are many subunit vaccine candidates, although none of them are directed at the pediatric population, given the history of ERD with subunit vaccines. These vaccines encompass SH, G, or F delivery and include a variety of adjuvants in their formulation [315].

In the case of hMPV, there are no vaccines or antibody treatments approved yet to treat or prevent the infection, but there are a few monoclonal antibodies and vaccine candidates with prophylactic potential [23,338]. These are organized in Table 10 and are briefly discussed below.

A vaccine candidate—that recently finished its phase 1 clinical trials—is a live attenuated recombinant hMPV expressing the P protein from the avian metapneumovirus (rHMPV-Pa). In this study, the vaccine was tested in infants that resulted in being seronegative for hMPV, indicating that this vaccine candidate was not immunogenic enough in this cohort [339]. Another vaccine candidate has recently began phase 1 clinical trials, consisting of two different mRNA sequences that express the F protein from two viruses, hMPV and parainfluenza virus type 3, which was created in lipid nanoparticles (mRNA-1653) [340]. Previous studies have indicated that this prototype is capable of inducing a proper immune response, thus making it a good vaccine candidate.

Additionally, it is important to mention that vaccine candidates against hMPV are continually being proposed. Among these highlights, it can be found a recombinant BCG expressing the P protein of hMPV (rBCG-P), which so far has demonstrated—in pre-clinical models—that it promotes viral clearance without inducing tissue damage [319].

As for prophylactic approaches against hMPV, mAbs are a good way to protect against this virus and to reduce the most severe symptoms. The antibody 54G10 is a human mAb which was compared to palivizumab, as it targets the F protein of hMPV. This mAb was tested in a mice model and the results demonstrated a significant neutralizing effect along with inducing a reduction in the viral titers in the lungs [341,342]. Even more, the human mAb 54G10 was able to decrease the viral titers from hMPV better than palivizumab did during hRSV infections. Another human mAb is the Fab DS7, which targets the F protein of hMPV. Fab antibodies are generated based on heavy and light chain genes that create a library of phage that are able to be combined. Even though Fabs are not common mAbs, Fab DS7 shows neutralizing effects. Unlike the previous mAb, this one was tested in cotton rats and the results proved to limit virus propagation in the lungs [341,343].

## 6. Conclusions

The innate immune response against respiratory viral pathogens can either be protective or contribute to damage against the lung epithelium. Even though hRSV and hMPV come from the same virus family and share extensive genomic similarities, along with having similar entry mechanisms into epithelial cells, they produce different innate immune responses.

The hRSV infection leads to the recruitment of DCs, AMs, neutrophils, eosinophils, NK cells, ILCs, and the activation of the complement system. Among these cells, AMs play a protective role against the infection with hRSV, while DCs can be protective—as seen for IFN-I-secreting pDCs—or detrimental, especially when infected by hRSV—as seen for T_H_2-promoting cDCs. The roles of neutrophils and eosinophils are controversial, due to some reports suggesting they have a protective antiviral role, while others propose they contribute to lung damage. Finally, NK cells, ILC2s, and the excessive activation of the complement system contribute to the pathogenesis of hRSV through uncontrolled inflammatory mechanisms. The respiratory epithelium is involved in the recruitment of innate immune cells, which contribute to the symptoms of hRSV infection. On the other hand, the hMPV infection leads to the recruitment of DCs, AMs, neutrophils, eosinophils, and NK cells. Among these cells, DCs have a protective role during hMPV infection. On the contrary, AMs contribute to damage caused in the lung epithelium. The role of neutrophils is controversial due to some reports suggesting that they have a protective role, while others propose they contribute to lung damage. Lastly, the protective role of eosinophils and NK cells remains to be studied, along with the possible contribution to the inflammation that ILCs and the complement system might have, which could be induced during the infection. The respiratory epithelium plays a role in the recruitment of these innate immune cells, contributing to the symptoms of hMPV infection.

The continuous efforts to develop a vaccine that can protect the population at risk against these viruses are important, in order to prevent at-risk groups from getting sick or generating severe symptoms caused by the immune response against these respiratory viruses. Certainly, an adequate vaccine candidate should elicit a proper innate immune response that can balance out the excessive inflammation that characterizes infection by these viruses, while still inducing protective and adequate adaptive immune responses.

## Figures and Tables

**Table 1:** Role of DCs in the pathology caused by hRSV and hMPV.

Study Model	hRSV	hMPV
In vivo model	○ FcγRIII receptor contributes to hRSV pathogenesis, possibly by enhancing DC infection. ○ Infected DCs can promote airway obstruction, enhance disease, and promote more severe allergic responses. ○ A low cDC1:cDC2 ratio correlates with enhanced disease severity.	○ The secretion of IL-12p40 of infected pDCs might contribute to the protection of the lungs.
In vitro model	○ One of the main sources of IFN-I. ○ Secretion of T_H_2-polarizing (IL-6, IL-10, IL-33) and proinflammatory cytokines (IL-1β, TNF-α), and poor CD4^+^ T cell activation. ○ Neonate DC subsets secrete less IFN-I, possess an impaired capacity to activate CD4^+^ T cells, and elicit different epitope-specific CD8^+^ T cell responses during the infection.	○ Main immune cell population to sense this virus. ○ hMPV decreases the CD4^+^ T cell activation and, as a consequence, it reduces the T_H_1 response. ○ DCs are capable of secreting IFNs, promoting the antiviral response.

**Table 2:** Role of AMs in the pathology caused by hRSV and hMPV.

Study Model	hRSV	hMPV
In vivo model	○ Contribute to disease resolution and ameliorate symptoms. ○ Controversial secretion of T_H_2-polarizing IL-33 and anti-inflammatory IL-10 at early time-points of infection.	○ Contributes to the pathogenesis of the infection. ○ Promotes the spreading of the virus in the airway epithelium.
In vitro model	○ One of the main sources of IFN-I upon RNA sensing. ○ Balanced early secretion of proinflammatory cytokines (IL-6, IL-8, and TNF-α) Abortive infection can occur.	○ One of the main sources of IFN-I. ○ The secretion of IL-1α/β can generate an inflammatory pathological response in the lungs.

**Table 3:** Role of neutrophils in the pathology caused by hRSV and hMPV.

Study Model	hRSV	hMPV
In vivo model	○ NETosis and ROS production may contribute to lung damage. ○ May stimulate excessive mucus production.	○ The extreme amount of neutrophils in samples of bronchioli and alveolar spaces correlates with the damage caused in the lung. ○ Lack of neutrophils aggravates the symptoms of the illness.
In vitro model	○ Extensive IL-8-mediated neutrophil infiltration to the airways during the infection. ○ No direct implication in virus clearance.	○ Migrating neutrophils express higher levels of CD11b and myeloperoxidase, promoting considerable damage to the epithelial cell monolayer.

**Table 4:** Role of eosinophils in the pathology caused by hRSV and hMPV.

Study Model	hRSV	hMPV
In vivo model	○ Eosinophilia is common among hRSV-ALRTIs. ○ Contribute to pulmonary infiltration of immune cells without direct lung damage, although EETs could be implicated in tissue damage.	○ Eosinophils cells are a parameter for the characterization of wheezing in patients infected with hMPV.
In vitro model	○ It can be infected, which induces secretion of T_H_2-polarizing cytokines. ○ Might possess antiviral activities.	○ The infection with this virus promotes the recruitment of eosinophils to the lungs.

**Table 5:** Role of NK cells in the pathologies caused by hRSV and hMPV.

Study Model	hRSV	hMPV
In vivo model	○ One of the last innate subsets to be recruited to the lung. ○ Early secretion of IFN-γ promotes detrimental lung inflammation.	○ Depletion with anti-CD49b/Pan-NK cells does not affect viral load. ○ Depletion with anti-NK1.1 does affect viral load. ○ NCR1 is an important receptor to control the infection.
In vitro model	○ Antiviral activity is controversial.	○ No information reported

**Table 6:** Role of ILCs in the pathology caused by hRSV.

Study Model	hRSV
In vivo model	○ Elevated levels of ILC2s correlate with a higher disease severity. ○ ILC2s enhance immunopathology through secretion of IL-13, T_H_2 polarization, and AHR enhancement upon activation through TSLP or uric acid.

**Table 7:** Role of the complement system in the pathology caused by hRSV.

Study Model	hRSV
In vivo model	○ Complement fixation through the classical pathway promotes the protection against the virus. ○ Anaphylatoxins C5a and C3a mediate increased AHR and lung infiltration of immune cells.
In vitro model	○ Complement fixation supports protective humoral responses and might exert antiviral activities via the alternate pathway as well.

**Table 8:** Cytokine secretion by epithelial cells during hRSV and hMPV infection.

Function	hRSV	hMPV
Angiogenesis, vasodilation, and endothelium activation	VEGF, FGF, IL-1α/β	IL-1α
Local and general inflammation	IL-1α/β, IL-6, TNF-α	IL-6, IL-32
Antivirals	IFN-λ, TRAIL	IFN-α, IFN-λ
Colony-stimulating factors	G-CSF, GM-CSF	-
T_H_2-polarizing cytokines	TSLP, HMGB-1, CCL2, IL-25, IL-33	TSLP, IL-33
DC, monocyte, and macrophage recruitment	CCL2, CCL3, CCL4, CCL5, CCL7	CCL2, CCL3, CCL4, CCL5
Neutrophil recruitment	CXCL8 (IL-8)	CXCL8 (IL-8), CCL5 (RANTES)
Eosinophil recruitment	CCL5, CCL11 (Eotaxin-1)	CCL5 (RANTES)
NK cell recruitment	CCL3, CCL4, CCL5, CXCL9, CXCL10, CXCL11	CCL3, CCL4, CCL5, CXCL10

**Table 9:** Ongoing studies of prophylactic treatments and vaccine candidates against hRSV.

Prophylactic Approach	Type	Name	Current Clinical Trials	NCT Number	References
Monoclonal Antibodies	Targeting F protein	Palivizumab	Market-approved	n.a.	[304,306,307,321,322]
		Motavizumab	Completed Phase 3	NCT00121108	[310,311,312]
				NCT00129766	
		MEDI8897	Ongoing Phase 3	NCT03959488	[313,314]
	Targeting N protein	Monoclonal anti-N antibody	Preclinical studies only	n.a.	[16]
Vaccine Candidates	Live-attenuated	RSV LID cpΔM2-2	Completed Phase 1	NCT02890381	[316]
				NCT02948127	
		RSV LID ΔM2-2 1030s	Completed Phase 1	NCT02952339	[323]
		RSV D46/NS2/N/ΔM2-2-HindIII	Completed Phase 1	NCT03099291	[324]
				NCT03102034	
		RSV-ΔNS2 Δ1313/I1314L	Ongoing Phase 1	NCT01893554 NCT03227029	[317,318]
		SeVRSV	Ongoing Phase 1	NCT03473002	[325,326]
	Live-attenuated/Chimeric	rBCG-N-hRSV	Ongoing Phase 1	NCT03213405	[319,320]
	Vector	VXA-RSVf	Ongoing Phase 1	NCT02830932	[16]
		MVA-BN-RSV	Ongoing Phase 2	NCT02873286	[327]
		ChAd155-RSV	Ongoing Phase 1	NCT03636906	[328]
			Ongoing Phase 2	NCT02927873	
		Ad26.RSV.PreF	Completed Phase 2	NCT03334695	[329]
			Ongoing Phase 2	NCT03982199	
	Particle	RSV F Nanoparticle	Completed Phase 1 (pediatric)	NCT02296463	[330,331,332]
			Completed Phase 3 (elderly)	NCT02608502	
			Completed Phase 3 (maternal)	NCT02624947	
	Subunit	DPX-RSV-SHe	Completed Phase 1	NCT02472548	[333]
		RSV F DS-Cav1	Completed Phase 1	NCT03049488	[334,335,336]
		GSK RSV F	Completed Phase 2	NCT02360475	[337]
				NCT02753413	
				NCT02956837	
		RSV F subunit vaccine (Pfizer)	Ongoing Phase 2	NCT03529773	[16]

**Table 10:** Ongoing studies of prophylactic treatments and vaccine candidates against hMPV.

Approaches	Type	Name	Current Clinical Trials	NCT Number	Reference
Prophylactics	Monoclonal antibody	54G10	Pending *	--	[342]
		Fab DS7	Pending *	--	[343]
Vaccine Candidates	Particle	mRNA- 1653	Completed Phase 1	NCT03392389	[340]
	Live-attenuated/Chimeric	rHMPV-Pa	Ongoing Phase 1	NCT01255410	[339]
		rBCG-P	Pending *	--	[319]

* Pre-clinical trial so far (animal models).
